# Evaluation of anxiety symptoms among caregivers of children that take therapy in the national center for children rehabilitation and treatment during COVID-19 pandemic

**DOI:** 10.1192/j.eurpsy.2021.774

**Published:** 2021-08-13

**Authors:** F. Dobi, B. Zenelaj, E. Kabili, A. Shtylla, D. Malaj, A. Demaj

**Affiliations:** Child And Adolescent Psychiatry, National Center for Children Rehabilitation and Treatment, Tirana, Albania

**Keywords:** COVID-19, caregivers, developmental disorder, Anxiety

## Abstract

**Introduction:**

Compared to the parents of kids with “typical” development the stress level and exhaustion in these parents is higher and more frequent. Furthermore COVID-19 pandemic can increase stress levels especially among people that suffer from mental health disorders. Studies show that these difficult, challenging times have had a negative impact on most families, which have a child with neurodevelopmental disorders.

**Objectives:**

Evaluation of different aspects of emotional dimension among caregivers of children that take therapy in the National Center for Children Rehabilitation and Treatment (NCCRT) during COVID-19 pandemic

**Methods:**

The study was conducted during a two-month period March-April 2020. The sample involved 110 relatives of children that were taking educative and rehabilitation therapy in NCCRT during last year, ambulatory or inpatients. Data were collected by clinical records and phone interviews. Instrument we used were: Demographic inventory and Hamilton Anxiety Rating Scale for anxiety symptom evaluation. All data were statistically analyzed through excel.

**Results:**

Most of individual interviewed, whom are responsible for children wellbeing and pass most of the time with them were their parents, 69% of them. 56% of individuals were among 31-45 years old and 92% of them were women. Physical distancing seemed to risen anxiety levels in caregivers whom hadn’t have these problems before.

**Conclusions:**

It is necessary the dynamic support with special attention for caregivers whom have emotional distress. Yet has to be evaluated the connection, if it’s present, between parents with emotional distress and children progress, for ones that are being supported with development therapy.

